# Intestinal Dysbiosis in Subjects with Obesity from Western Mexico and Its Association with a Proinflammatory Profile and Disturbances of Folate (B9) and Carbohydrate Metabolism

**DOI:** 10.3390/metabo14020121

**Published:** 2024-02-11

**Authors:** Verónica Riggen-Bueno, Susana Del Toro-Arreola, Tonatiuh Abimael Baltazar-Díaz, Alejandra N. Vega-Magaña, Marcela Peña-Rodríguez, Paula Alejandra Castaño-Jiménez, Laura Verónica Sánchez-Orozco, José María Vera-Cruz, Miriam Ruth Bueno-Topete

**Affiliations:** 1 Servicio de Nutrición Clínica, Hospital Civil de Guadalajara, Unidad Hospitalaria Fray Antonio Alcalde, Hospital 278, Guadalajara CP 44280, Jalisco, Mexico; vriggen@hcg.gob.mx; 2 Maestría en Nutrición Clínica, Universidad del Valle de Atemajac, Tepeyac 4800, Zapopan CP 45050, Jalisco, Mexico; 3 Instituto de Investigación en Enfermedades Crónico Degenerativas, Departamento de Biología Molecular y Genómica, Centro Universitario de Ciencias de la Salud, Universidad de Guadalajara, Sierra Mojada 950, Guadalajara CP 44340, Jalisco, Mexico; susana@cucs.udg.mx (S.D.T.-A.); paula.castano3083@alumnos.udg.mx (P.A.C.-J.); laura.sorozco@academicos.udg.mx (L.V.S.-O.); 4 Instituto de Investigación en Ciencias Biomédicas, Departamento de Clínicas Médicas, Centro Universitario de Ciencias de la Salud, Universidad de Guadalajara, Sierra Mojada 950, Guadalajara CP 44340, Jalisco, Mexico; alejandra.vega@academicos.udg.mx; 5 Laboratorio de Diagnóstico de Enfermedades Emergentes y Reemergentes, Departamento de Microbiología y Patología, Centro Universitario de Ciencias de la Salud, Universidad de Guadalajara, Sierra Mojada 950, Guadalajara CP 44340, Jalisco, Mexico; marcela.pena@cucs.udg.mx; 6 Instituto de Nutrigenética y Nutrigenómica Traslacional, Departamento de Biología Molecular y Genómica, Centro Universitario de Ciencias de la Salud, Universidad de Guadalajara, Sierra Mojada 950, Guadalajara CP 44340, Jalisco, Mexico; jose.vcruz@academicos.udg.mx

**Keywords:** gut microbiota, obesity, western Mexico, metagenomic prediction, 16S rRNA sequencing

## Abstract

Obesity is a public health problem with a growing prevalence worldwide. In Mexico, it is estimated that one out of three adults suffer from obesity. In these patients, the intestinal microbiota (IM) undergoes pathological changes that are associated with a dysbiotic state; however, the microbiota profile of adult subjects with obesity from western Mexico has not been described. To assess this, fecal samples were obtained from 65 participants (Obese = 38; Control = 27). The microbial composition was characterized by 16S rRNA amplicon sequencing. The IM of the group with obesity revealed a clear decrease in richness and diversity (*p* < 0.001), as well as a significant increase in proinflammatory bacterial groups, mainly genera belonging to the Negativicutes class, *Escherichia*/*Shigella*, and *Prevotella*. Likewise, an increase in short-chain fatty acid-producing bacteria was found, especially the genus *Lachnoclostridium*. Additionally, PICRUSt2 analysis showed a depletion of vitamin B9 metabolism and an increase in saccharolytic pathways. The IM of patients with obesity possesses a dysbiotic, proinflammatory environment, possibly contributing to lipogenesis and adiposity. Thus, assessing the IM will allow for a better understanding of the pathophysiology of metabolic diseases of high prevalence, such as obesity. These findings are described for the first time in the adult population of western Mexico.

## 1. Introduction

Obesity is one of the most prevalent health problems in the world. It is defined as a chronic, multifactorial metabolic disorder resulting from the interaction between genetics and the environment. According to the World Health Organization (WHO), the prevalence of obesity has almost tripled worldwide since 1975. It is estimated that more than 1.9 billion adults (39% of the adult population) are overweight [1]. Of these, 650 million are classified as obese, defined by a body mass index (BMI) that is greater than or equal to 30 kg/m^2^. If these trends continue, by 2025, the global prevalence of this disease will be 18% in men and will exceed 21% in women [1]. In the case of Mexico, 70% of Mexicans are currently overweight, and approximately one-third of the population suffers from obesity [2]. Obesity is associated with multiple comorbidities, including cardiovascular disease (CVD), metabolic syndrome (MetS), and cancer [1].

Obesity is also a major risk factor for mortality; in fact, it is estimated that 3 million adults die each year from this cause. After tobacco, it is considered the second most preventable cause of premature death in the world [3]. In Mexico, a sedentary lifestyle and an unhealthy diet are responsible for 20% of deaths in men and 32% in women [2].

In the last decade, a promising field of research has focused on the connection between the intestinal microbiota (IM) and human health. The IM is a complex community of microorganisms that colonize the human gastrointestinal tract. The number of microbes in the IM can be as high as 100 trillion, and it contains approximately 100 times more genes (microbiome) than there are in the human genome [4]. IM plays a relevant role in various physiological processes in the body, including immune function, digestion, and insulin sensitivity, as well as in neurological and behavioral functions. Several factors can modify the gut microbiota, including host genetics, diet, age, mode of birth (cesarean or vaginal), and antibiotic use [5].

The gut microbiota is especially sensitive to changes in dietary habits. In fact, it has been suggested that 60% of the gut’s microbial composition is determined by the host’s diet [6]. As an example, the Mediterranean diet (MD) is well known for its positive impact on the IM’s α-diversity and metabolic activities [7]. The Mediterranean microbiota protects against CVD and tumor pathologies due to its production of more SCFAs by fiber-degrading bacteria [8]. Moreover, plant protein and high-fiber regimens, such as the MD, stimulate the growth of probiotic species like *Bifidobacteria*, which are commonly inhibited under a Western-style diet [9].

Advances in 16S rRNA gene sequencing techniques have contributed to a better understanding of the IM and its association with different pathophysiological conditions. Dysbiosis is defined as a pathological modification of the gut microbiota (both structural and functional), making it capable of inducing systemic inflammation through the increase in proinflammatory bacterial communities [10]. Some diseases that are associated with pathological disturbances of IM are inflammatory bowel disease, diabetes, allergies, autoimmune diseases, cardiovascular disease, hypertension, and, in particular, obesity [5]. In fact, the composition of this bacterial community appears to be involved in energy metabolism, playing an important role in the etiology and pathophysiology of obesity [11].

The association of gut bacteria and obesity was demonstrated by pioneering studies in germ-free mice. On the one hand, Farias et al. demonstrated that conventional mice have 47% more adipose tissue than germ-free mice. Subsequently, a fecal transplantation from a conventional to a germ-free mouse showed a 60% increase in adipose tissue, as well as insulin resistance, and increased levels of leptin and serum glucose [12]. The composition of the microbial profile appears to be different when compared between subjects with and without obesity. Changes in almost all taxonomic categories have been reported in the global literature, mainly at the phylum, family, genus, and even species level. It has also been observed that individuals with a high percentage of body fat tend to show changes in the diversity and richness of the IM, as well as a higher amount of proinflammatory bacteria [4]. These findings demonstrate the relevance of the IM to body composition, as well as to clinical parameters related to metabolic dysfunction [1].

Studies on IM in subjects with obesity have concluded that people with a high BMI differ in their microbial profile compared to individuals with a lower BMI. At the genus level, lower proportions of *Bifidobacterium* and *Eggerthella* and higher proportions of *Dorea*, *Escherichia*/*Shigella*, *Eubacterium*, *Fusobacterium*, *Megasphera*, *Prevotella*, *Roseburia*, *Streptococcus*, and *Sutterella* have been described at higher BMIs [13]. Higher relative abundances of the phyla Firmicutes, Proteobacteria, and Fusobacteria, as well as the genus *Lactobacillus*, have also been described in individuals with obesity. On the other hand, species such as *Faecalibacterium prausnitzii*, *Akkermansia muciniphila*, and *Methanobrevibacter smithii* were found in lower relative abundances in adults with a high body weight compared to lean individuals [11,14]. However, systematic reviews on IM and obesity agree that the only thing that they have in common is the heterogeneity of their results. While some bacterial taxa may be associated with the obese phenotype in one population, they may be characteristic of lean individuals in a different region. This reflects the striking individuality of this bacterial community; it also demonstrates its sensitivity to environmental, genetic, and lifestyle factors [15].

In the population of western Mexico, to this date, there is no published research work that describes the IM profile of adult subjects with obesity. Therefore, the present work aims to compare the IM profile of adults with and without obesity in this region. These findings will enable a better understanding of the pathophysiology of obesity, which may lead to the development of therapeutic targets that could be implemented in the future.

## 2. Materials and Methods

### 2.1. Study Design

This cross-sectional, comparative study was carried out in the Institute of Chronic Degenerative Diseases of the University of Guadalajara. Sixty-five volunteer male and female participants were included in the study. They were recruited from the metropolitan area of the city of Guadalajara through a first-hand invitation from the researchers. Thirty-eight subjects were included in the obesity (Ob) group and twenty-seven in the control group. The recruitment time took place from May 2022 to November 2022. All participants signed an informed consent form, and this study was conducted in accordance with the guidelines of the World Medical Association (Declaration of Helsinki, revised in 2013).

Anthropometric characteristics, particularly BMI, were used to classify participants into the obesity (Ob) or the control groups. Inclusion criteria for patients with obesity were (a) BMI > 29.9 kg/m^2^, (b) age between 18 and 59 years, (c) Mexican citizens (born in Mexico), (d) current residents of a western Mexican state (Colima, Jalisco, Michoacán, and Nayarit), (e) not currently undergoing a nutritional intervention for weight loss, (f) no current or past SARS-CoV-2 infection for at least 30 days before recruitment, (g) no current or past use of prebiotics/probiotics for at least 30 days before recruitment, (h) no current or past use of antibiotics at least 30 days before recruiting.

Inclusion criteria for subjects without obesity (control group) were (a) BMI < 29.9 kg/m^2^, (b) age between 18 and 59 years, (c) Mexican citizens (born in Mexico), (d) current residents of a western Mexican state (Colima, Jalisco, Michoacán, and Nayarit), (e) not currently undergoing a nutritional intervention for weight loss, (f) no current or past SARS-CoV-2 infection for at least 30 days before recruitment, (g) no current or past use of prebiotics/probiotics for at least 30 days before recruitment, (h) no current or past use of antibiotics at least 30 days before recruitment.

### 2.2. Anthropometric Evaluation and Biochemical Parameters

Body weight and body fat percentage were measured under fasting conditions using a bioelectrical impedance scale (OMRON HBF-514C Body Composition Monitor with Scale). Height was measured using a wall-mounted stadiometer. Measurements were taken in the standing position after removing shoes. Basal biochemical data were obtained from a venous sample, also under a 12 h fasting condition. The biochemical data obtained were the following: glucose, triglycerides (TG), total cholesterol (TC), high-density lipoprotein cholesterol (HDL-c), and low-density lipoprotein cholesterol (LDL-c).

### 2.3. Blood Pressure

Systolic and diastolic blood pressures were obtained using a blood pressure monitor (OMRON HEM-7120). The cuff was placed and tightened on the patient’s left arm while in a resting position.

### 2.4. Extraction of Nucleic Acids and 16S rRNA Amplicon Sequencing

Fecal samples were collected and immediately stored at −80 °C. DNA was extracted from 150 mg of frozen feces with QIAamp PowerFecal DNA Kit (QIAGEN, Hilden, Germany) according to the manufacturer’s protocol. DNA was quantified with NanoDropTM OneC spectrophotometer (Thermo Scientific, Waltham, MA, USA).

The 16S metagenomic sequencing library preparation was performed according to Illumina MiSeq System’s protocol (Illumina, San Diego, CA, USA) [16]. V3 and V4 regions from 16S were amplified with Platinum Taq DNA Polymerase High fidelity (Invitrogen, Carlsbad, CA, USA) using primers with adaptors. The sequence of the primers used were as follows: Forward: (5′TCGTCGG-CAGCGTCAGATGTGTATAAGAGACAGCCTACGGGNGGCWGCAG-3′), reverse: (5′GTCTCGTGGGCTCGGAGATGTGTATAAGAGACAGGACTACHVGGG- TATCTAATCC-3′). PCR conditions were followed according to protocol. Product purification was achieved with AMPure XP^®^ (Beckman Coulter, Brea, CA, USA) magnetic beads and was quantified with Qubit^®^ 3 dsDNA HS kit (Invitrogen, Carlsbad, CA, USA) according to product indications (Supplemental Figure S3). Next, index incorporation was achieved with Nextera XT Index Kit v2 Set A (No. Cat. FC-131-2001, Illumina, San Diego, CA, USA) by a second PCR amplification. Finally, amplicons were pooled to equimolar concentrations into a 4 nmol/L solution tube, library denaturing, and MiSeq Sample Loading (kit Miseq Reagent V3 600-cycle, Illumina, San Diego, CA, USA) according to protocol.

### 2.5. Bioinformatic Analysis of 16S Amplicon Sequencing

Microbiome bioinformatics were performed with QIIME2 version 2023.2 [17]. Sequences whose quality parameter was Phred ≥ 30 were filtered by denoising with DADA2 via q2-dada2 [18]. All resulting amplicon sequence variants (ASVs) were aligned with MAFFT [19] (via q2-alignment) and used to construct a phylogeny with FastTree2 [20] (via q2-phylogeny). Taxonomy was assigned to ASVs using a pre-trained classifier against the Silva 138 full-length sequences database [21,22,23]. ASVs identified as mitochondria and chloroplasts were removed from the feature table. Alpha diversity metrics (observed features, Shannon and Chao1 indices) [24], as well as beta diversity metrics (Weighted UniFrac and unweighted UniFrac) [25] and Principal Coordinate Analysis (PCoA) were generated and tested with QIIME2 pipeline. Linear discriminant analysis effect size (LEfSe) was obtained with the Galaxy interface [26,27]. The threshold cutoff value of the LDA score was 3.1. Phylogenetic Investigation of Communities by Reconstruction of Unobserved States (PICRUSt2) pipeline [28,29,30,31,32] was used to predict the functional pathways of each group according to the MetaCyc Database [33], whose results were then analyzed using LEfSe with a threshold of 1.8. ANCOM-BC (analysis of the composition of microbiomes with bias correction), which is a compositionally based method [34], was performed at the family and genus level to determine differentially abundant taxa using the q2-composition plugin, implemented in the QIIME2 pipeline.

To calculate Proteobacteria/Firmicutes ratio, a centered log-ratio (clr) transformation was applied to feature table at phylum and genus levels through MicrobiomeAnalyst server [35]. Then, normalized ratios of taxa identified as Firmicutes or Proteobacteria were calculated as previously described [36]. For Gram-positive/Gram-negative ratio, taxa were filtered from raw ASV table collapsed at the species level. All taxa that could not be identified at the genus or species level were removed, as well as those with putative names or uncultivated species, obtaining a filtered abundance table. Gram staining was examined according to BacDive database [37]. Undetermined or variable Gram staining taxa were removed. Undetermined or variable Gram staining taxa were removed. In case the genus or specie were not in the BacDive database, the primary publication of each genus or specie was examined. Using this same filtered abundance table, oxygen tolerance was evaluated with information from the BacDive database, complemented by public databases (https://www.mediterranee-infection.com/wp-content/uploads/2020/05/OXYTOL-1.3.xlsx, accessed on 28 December 2021), as described by Dubourg et al. [38]. Obligate aerobes, microaerophiles, and facultative anaerobes were considered aerobes, whereas only obligate anaerobes were considered true anaerobes, according to Kumar et al. [39].

### 2.6. Statistical Analyses

When assessing the participants’ background characteristics, Student’s *t*-test or U of Mann–Whitney test were employed, depending on parametric or non-parametric variables. Chi-squared test was used when assessing categorical variables. Alpha diversity medians were compared using Mann–Whitney U test. Beta diversity metrics among groups were statistically analyzed by performing PERMANOVA tests. Both alpha and beta diversity statistical analyses were corrected with Benjamini–Hochberg (BH) multiple testing through QIIME2 package. Spearman’s rank correlation between selected taxa and biochemical values was performed. All statistical tests were two-sided, and a *p*-value or false discovery rate-adjusted *q*-value of less than 0.05 were considered statistically significant. Data were analyzed using SPSS 25.0, unless otherwise specified. Plots were generated utilizing GraphPad Prism version 9.0.0.

## 3. Results

### 3.1. Cross-Sectional Study and Clinical Assessments

In this study, the total cohort was sixty-five participants, of which thirty-five were women (53.8%) and thirty were men (46.2%), with an average age of 37.7 ± 12.0 years. Thirty-eight of them had a BMI that was greater than 30 kg/m^2^; therefore, they were assigned to the Ob group. Of these thirty-eight subjects, 44.7% were classified as class I obesity, 21.05% as class II, and 34.21% as class III. The remaining twenty-seven participants were assigned to the control group (BMI < 30 kg/m^2^). There were no significant differences in the age and sex of the participants between both groups. However, as expected, the BMI and body fat percentage of the obesity (Ob) group were significantly higher compared to the control (*p* < 0.001). Additionally, blood parameters that assess metabolic function (fasting glucose, HDL-c, TG, TC), as well as blood pressure, did show significant differences between groups (Table 1).

### 3.2. Microbiota Diversity between Groups

**Figure 1:**
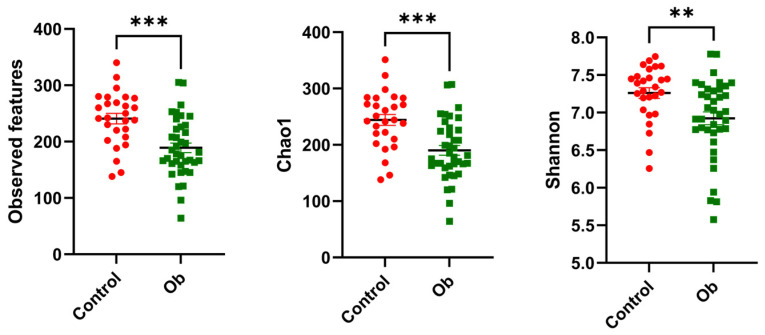
Gut microbiota richness and diversity. Dot plots showing alpha diversity indices that account for richness (observed features), low-abundance species (Chao1), and diversity (Shannon), among subjects without obesity (control) and subjects with obesity (Ob). Individual sample values are shown as red and green dots, respectively. Lines inside the dots represent median values. Medians were compared with Mann–Whitney U test, ** *p* < 0.01; *** *p* < 0.001.

The alpha diversity was calculated using observed features (analogous to observed ASVs) and Shannon and Chao1 indices. The observed feature analysis expresses the species richness in a community, Shannon estimates the richness and diversity, and Chao1 estimates the diversity based on abundance (Figure 1). We observed a significant decrease in the three indices within the Ob group compared with the control group (Observed features and Chao1, *p* < 0.001; Shannon, *p* = 0.003).

**Figure 2:**
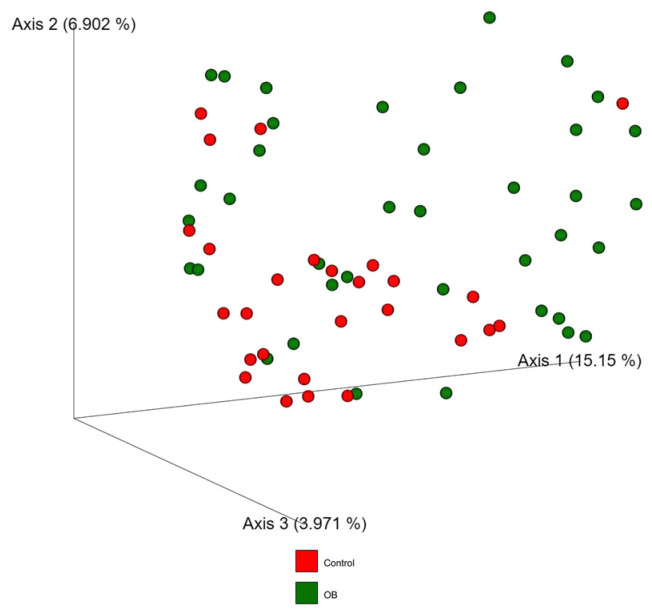
Bacterial beta diversity. The figure shows a three-dimensional scatter plot, using PCoA from unweighted Unifrac analyses. The plot shows the distances between the bacterial profiles of subjects with obesity (green spheres) and without obesity (red spheres). Statistical analyses and *p*-value calculation were performed with PERMANOVA test with Benjamini–Hochberg (BH) multiple testing correction. *p* < 0.001.

The beta diversity analysis was evaluated by unweighted UniFrac metrics, in order to show how similar or different the bacterial diversity between both groups was (Figure 2). Results were plotted by Principal Coordinate Analysis (PCoA). We clearly observed the conformation of two well-defined groups in the unweighted UniFrac plot, which implies an evidently different microbiome profile between the participants with and without obesity (PERMANOVA, *p* < 0.001).

### 3.3. Relative Abundances

At the phylum level, the Proteobacteria/Firmicutes (P/F) ratio was significantly increased in the Ob group compared to the control (*p* < 0.05) (Supplemental Figure S1). The Firmicutes/Bacteroidetes ratio did not show significant differences between the groups. Another relevant finding was the predominance of aerobic and Gram-negative bacterial groups, with respect to anaerobic and Gram-positive bacteria, in the Ob group compared to the control (*p* < 0.05, both ratios, Supplemental Figure S1).

### 3.4. Differential Abundances (LEfSe and ANCOM-BC)

**Figure 3:**
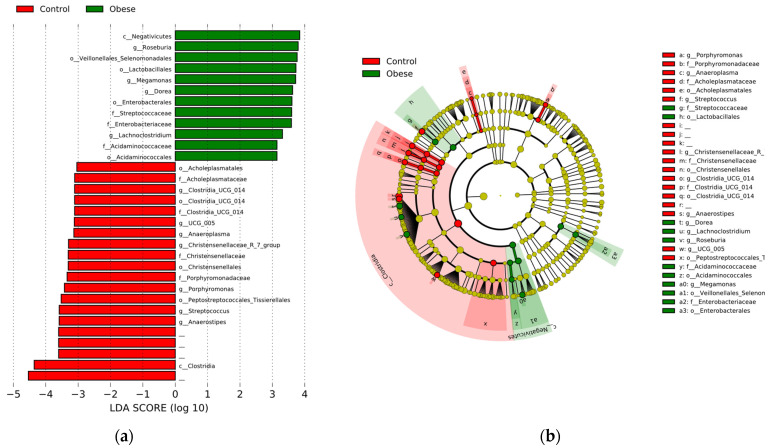
Linear discriminant analysis effect size (LEfSe) analysis to identify the differentially abundant taxa in patients with obesity (Ob) and without obesity (control). (**a**) Bar diagram. Green bars indicate taxa representative of the Ob group, while red bars represent the control group. The size of the bars represents the effect size (LDA) between groups (LDA > 3.10, *p* < 0.05). (**b**) Cladogram. Green circles indicate taxa characteristic of the Ob group, while the red circles represent the control group. Yellow circles represent taxa with no significant difference. Both graphs show the differentially abundant taxa at the phylum, class, order, family and genus level between the two groups.

The linear discriminant analysis (LEfSe) was used to determine the bacterial taxa that differentially characterize both study groups (Figure 3), i.e., which taxa are statistically representative of each group (LDA ≥ 3.10, *p* < 0.05), and which might explain the observed clinical differences between them. The analysis confirmed that bacterial members belonging to the Negativicutes class and the genus *Lachnoclostridium* are taxa that are associated with the Ob group. These bacteria are known for their production of short-chain fatty acids (SCFAs). In the case of Negativicutes, they are also distinguished by their proinflammatory potential, due to the presence of lipopolysaccharide (LPS) in the outer membrane of their cell wall. Other proinflammatory bacteria were also found in the Ob group, such as members of the Streptococcaceae family (order Lactobacillales) and Enterobacteriaceae. On the other hand, the control group was characterized by bacteria belonging to the Clostridia class, especially the families Christensenellaceae and Lachnospiraceae. In this group, some genera such as *Porphyromonas* and *Anaeroplasma*, as well as the Acholeplasmataceae family, were also enriched. Both groups were dominated by bacteria belonging to the phylum Firmicutes.

**Figure 4:**
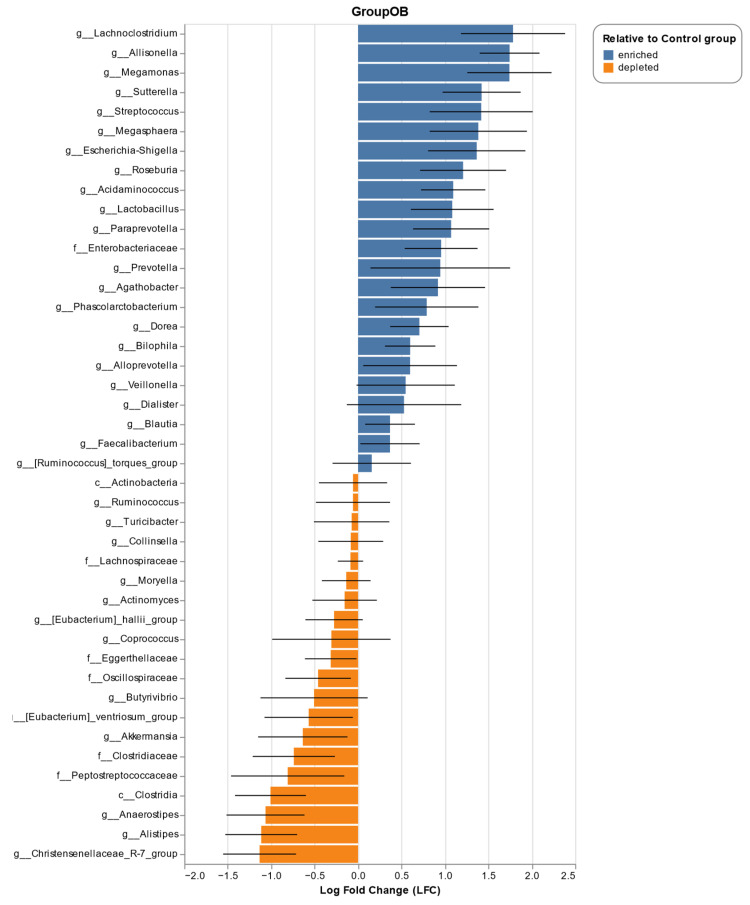
Analysis of Compositions of Microbiomes with Bias Correction (ANCOM-BC) to identify the differentially abundant taxa in patients with obesity (Ob) and without obesity (control). The bars represent the log fold change (LFC) between both groups. Blue bars indicate characteristic taxa of the Ob group, while brown bars represent the control group. Top 22 taxa ordered by LFC at family and genus level are shown. A threshold of *p* < 0.05 and *q* < 0.05 was employed.

To complement the LEfSe analysis, and to strengthen the results of the differential abundances, ANCOM-BC analysis was performed (Figure 4). This analysis has greater statistical power, as it has the ability to control for possible biases that are associated with false positives. Confirming the LEfSe findings, the ANCOM-BC results showed that several genera belonging to the Negativicutes class are significantly enriched in Ob patients. These genera include *Allisonella*, *Megamonas*, *Megasphaera*, *Acidaminococcus*, *Veillonella*, *Dialister*, and *Phascolarctobacterium*. In addition, the most representative genus of the Ob group was *Lachnoclostridium*. It was also found that members belonging to the phylum Proteobacteria, such as the genera *Escherichia*/*Shigella* and *Suterella*, as well as the family Enterobacteriaceae, were strongly associated with the Ob group. Other taxa that were characteristic of the obese phenotype were some genera belonging to the families Lachnospiraceae (*Dorea*, *Tyzzerella*, *Roseburia*, *Agathobacter*, *Blautia*), Prevotellaceae (*Prevotella*, *Paraprevotella*, and *Alloprevotella*), and Ruminocaccaceae (*Subdoligranulum*, and *Faecalibacterium*), as well as the order Lactobacillales (*Streptococcus*, *Lactobacillus*, *Weisella*, *Enterococcus*, and *Lactococcus*). In general terms, Gram-negative bacteria with proinflammatory potential were enriched in the Ob group, which is associated with metabolic dysfunction. However, important bacterial groups producing SCFAs, especially acetate, were also found to be enriched in subjects with obesity.

On the other hand, and in agreement with LEfSe, the control group was characterized by the presence of bacteria belonging to the Clostridia class, especially the families Christencencenellaceae, Oscillospiraceaceaceae, and Peptostreptococcaceae. The genera *Akkermansia* (phylum Verrucomicrobia) and *Eggerthella* (phylum Actinobacteria) were also found to be enriched. Likewise, members of the family Lachnospiraceae were representative of the control group, especially genera such as *Anaerostipes*, *Butyrivibrio*, *Coprococcus*, *Moryella*, and the species *Eubacterium halli*. Finally, other genera that were characteristic of this group were *Alistipes*, *Rombutsia*, *Colidextribacter*, *Desulfovibrio*, *Turicibacter*, and *Actinomyces*. Broadly speaking, it is evident that the control group possesses a greater diversity in terms of the families that make up the IM, as well as a significant predominance of Gram-positive SCFA-producing bacteria, especially butyrate, which has positive effects on intestinal health and energy metabolism [40].

### 3.5. Functional Prediction of Metabolic Pathways (PICRUSt2)

**Figure 5:**
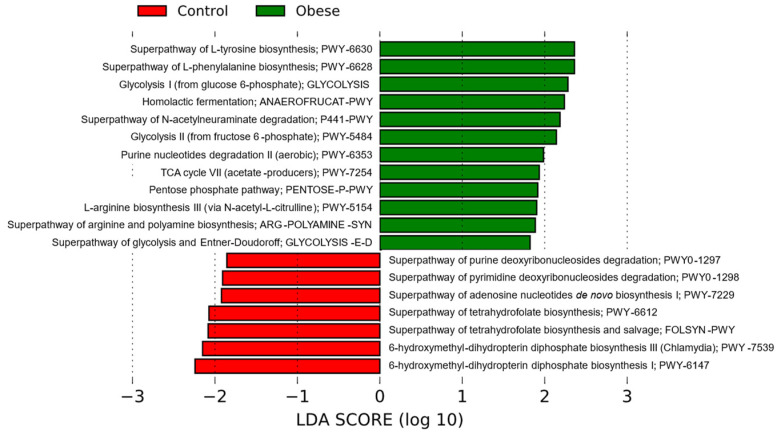
Predictive analysis of metabolic pathways by PICRUSt2 of the gut microbiota in patients with obesity (Ob) and without obesity (control). Metabolic pathways are represented in accordance with the MetaCyc database. The size of the bars represents the effect size (LDA) between study groups. The threshold of LDA > 1.8, *p* < 0.05 was applied. Green bars represent bacterial metabolic pathways enriched in the obese group, while red bars represent the control group. PICRUSt2 (Phylogenetic Investigation of Communities by Reconstruction of Unobserved States).

PICRUSt2 was used to infer the functional mechanisms of the intestinal microbiota, that is, which bacterial metabolic pathways are most prominent in each group, depending on the microbial profile. The results of this algorithm were analyzed by LEfSe analysis (LDA ≥ 1.8, *p* < 0.05) (Figure 5). Comparisons between groups revealed that the Ob group has increased metabolism of some amino acids, such as L-tyrosine, L-phenylalanine, and L-arginine. Some pathways of energy metabolism were also increased, such as glycolysis, the Krebs cycle, the pentose phosphate pathway, and homolactic fermentation. As for the control group, nucleic acid metabolism pathways (purine and pyrimidine degradation and adenosine biosynthesis) predominated. The synthesis pathways of some cofactors such as folates (vitamin B9) were also increased, which lead to the biosynthesis of methionine, purines, and pyrimidines. Of particular relevance, the obese group showed a depletion of vitamin B9 (folate) metabolism.

### 3.6. Correlation Analysis

We further employed Spearman’s correlations between specific taxa and blood biochemical parameters. Notably, we found significant negative correlations between the *Eubacterium hallii* group and blood parameters such as triglycerides (*p* < 0.01) and glucose (*p* < 0.01) In contrast, we observed positive correlations between the *Eubacterium hallii* group and HDL-c (*p* < 0.05) (Supplemental Figure S2).

## 4. Discussion

Obesity is a highly prevalent public health problem worldwide. This disease is a risk factor for developing other comorbidities such as type 2 diabetes mellitus (T2DM), CVD, and cancer. Recently, it has been discovered that there is a relationship between obesity and a dysbiosis of the IM, which could be involved in the etiology and pathophysiology of obesity [4]. The present work is a pioneer in describing the alterations of the gut microbiota in adult subjects with obesity in western Mexico.

Regarding the alpha and beta diversities of the IM, we found substantial differences between both study groups. In patients with obesity, we observed a significant reduction in bacterial diversity and richness (alpha diversity) compared with the control group. This low bacterial diversity has clinical relevance, as a reduction in this metric has been associated with dysbiosis, as well as with the presence of several acute and chronic diseases. This suggests the possible importance of a complex microbial composition in order to exert a true commensal relationship with the host [41]. It is important to mention that, regarding this metric, the results in different populations have been discordant. For example, Chávez-Carbajal et al. reported that the alpha diversity was higher in a population of women with obesity and MetS in Mexico City, compared to women with a lower BMI [42]. On the other hand, some meta-analyses confirm that in other countries, alpha diversities have been significantly lower in patients with obesity; among them are Japan, France, Saudi Arabia, and Egypt, as well the Latin American migrant population of the United States [13]. This reflects the marked variability in the findings of IM studies in different geographic and cultural contexts [15].

On the other hand, in terms of differential abundances, LEfSe and ANCOM analyses revealed a gut microbial profile with a highly proinflammatory and pathogenic potential in subjects with obesity. This profile is dominated by Gram-negative bacteria in a favorable aerotolerant environment, in accordance with the ratios previously analyzed, which were found to be statistically significant. In contrast, the bacterial profile found in the control group was dominated by Gram-positive bacteria in an anaerobic microenvironment. The results obtained from these ratios are novel findings, given that they have not yet been described in the context of obesity.

One of the taxa that was significantly increased in the Ob group with respect to the control were the Negativicutes. These are a rare class of Gram-negative bacteria, which correlate positively with LPS-induced proinflammatory pathways. It is worth noting that a high plasma concentration of this endotoxin is associated with a high-fat diet, which could cause local intestinal inflammation, increasing gut barrier permeability and the subsequent translocation of LPS into systemic circulation [43].

The main Gram-negative genera that we found, which belong to the Negativicutes class, were *Allisonella*, *Megamonas*, *Megasphaera*, *Acidaminococcus*, *Veillonella*, *Dialister*, and *Phascolarctobacterium*. In fact, these bacteria were practically absent in the participants without obesity in the present work. These genera have been poorly described in Latin American countries, and little is known about their role in the pathophysiology of obesity. However, we found that the Negativicutes could be a characteristic hallmark of the obese phenotype in the population with obesity of western Mexico. This class has been found to be enriched in other populations with obesity in China and with T2DM in Pakistan. In addition, it is positively associated with the presence of steatohepatitis [44,45]. Importantly, *Dialister* and *Phascolarctobacterium* (both members of the Negativicutes) have been positively related to BMI and body weight gain, respectively. The proinflammatory potential of the Negativicutes class is relevant, as it is known that a chronic, low-grade inflammation could lead to the development of insulin resistance and MetS [43,46]. A clear association has been described between inflammation and decreased insulin sensitivity, which causes increased free fatty acid efflux from adipocytes and ectopic fat deposition [47]. Additionally, chronic systemic inflammation has been found to increase lipogenesis in the liver. Proinflammatory cytokine TNF-α is thought to induce hepatic lipid accumulation through increased fatty acid uptake, enhanced TG synthesis, and reduced fatty acid oxidation. Therefore, chronic low-grade inflammation might have a direct effect on the development of ectopic fat deposition and lipogenesis [48].

Another proinflammatory bacterial group that we found to be significantly enriched in the population with obesity was the Enterobacteriaceae family (phylum Proteobacteria), as well as the genera *Escherichia*/*Shigella* and *Sutterella*. It is known that Enterobacteriaceae are capable of inducing a proinflammatory response through the release of LPS [49]. Anhe et al. found that subjects with obesity and T2DM have increased levels of this family in their microbiota, not only at the gastrointestinal level, but also in their plasma, liver, and adipose tissue [50]. Particularly, *Escherichia*/*Shigella* has been positively associated with insulin resistance, T2DM, and weight loss difficulty after a dietary intervention [51]. These findings are in agreement with the Proteobacteria/Firmicutes (P/F) ratio, which was significantly increased in the Ob group. This ratio has scarcely been investigated in microbial taxonomy studies; however, our research group recently reported that patients with HIV and MetS who were treated with integrase strand transfer inhibitors (INSTIs), compared to those treated with protease inhibitors (PIs), displayed a significantly increased P/F ratio. These changes were associated with a more profound dysbiosis [36]. Therefore, we suggest a deeper investigation of the P/F ratio in various pathologies, in order to better understand the changes that occur at the phylum level, as well as its possible clinical implications.

Along the line of Gram-negative bacteria with potential immunogenic effects, we found that some genera of the Prevotellaceae family were enriched in the Ob group; among them were *Prevotella*, *Paraprevotella*, and *Alloprevotella*. In fact, according to Xu et al., *Prevotella* has been described as a representative taxon of obesity-associated dysbiosis [15]. In addition, positive relationships have been reported between *Prevotella* and some metabolic disorders such as insulin resistance, hypertension, and non-alcoholic fatty liver disease (NAFLD) [52].

Another relevant finding regarding the IM profile that was found in patients with obesity was its remarkable association with the production of short-chain fatty acids (SCFAs). Members of the Negativicutes, for instance, are important producers of these metabolites. Below, we describe the bacterial genera that belong to this class and were enriched in the Ob group: the genus *Megamonas*, for example, possesses a metabolic pathway which produces acetate; this pathway promotes the accumulation of triglycerides, and the subsequent development of NAFLD [53]. *Veillonella*, on the other hand, is capable of producing acetate and propionate through pathways that involve lactate and butyrate [54]. Several studies have highlighted that acetate can induce obesity, as it stimulates the synthesis of fatty acids and cholesterol, as well as the storage of adipose tissue [55]. As for butyrate, it can be produced by *Megasphaera* using amino acids (glutamate and lysine) as substrates. It is hypothesized that a reduced intake of non-digestible carbohydrates leads to increased conversion of amino acids into SCFAs by the intestinal microbiota. It should be noted that these pathways generate ammonia, which can have deleterious effects on health [54].

Additionally, we found that some genera belonging to the family Lachnospiraceae were also enriched in the Ob group of our study (*Lachnoclostridium*, *Roseburia*, *Agathobacter*, *Dorea*, *Blautia*, and *Ruminococus torques*). Most of these bacteria are known to produce SCFAs via a pathway that utilizes acetyl-CoA [31]. Likewise, *Lachnoclostridium* has been positively associated with visceral fat, and this association is thought to be partially mediated by acetate. In addition to its contribution to energy harvesting through SCFAs, it is believed that *Lachnoclostridium* can increase levels of trimethylamine N-oxide (TMAO), which is a metabolite that is commonly associated with cardiometabolic disorders, including obesity and T2DM. Similarly, some species of this genus have metabolic pathways belonging to the lipid biosynthesis category [56].

Our findings are in agreement with Palmas et al., who describe an SCFA-producing microbial profile being associated with the obese phenotype in Italy. A possible explanation is that the fermentation of non-digestible polysaccharides to SCFAs may induce hepatic lipogenesis and triglyceride accumulation in host adipocytes. Although SCFAs are generally known to have beneficial effects on host metabolism and body weight, this process could be impaired in obesity, especially in the context of a high intake of carbohydrate-rich foods, which increases energy harvesting from the diet and leads to hepatic lipogenesis [54].

Conversely, the bacterial taxa that we found to be enriched in the control group are associated with parameters that indicate host metabolic homeostasis and are representative of the lean phenotype. Among them is the Christensenellaceae family, which has been positively associated with a normal BMI (18.5–24.9 kg/m^2^) and with low levels of adiposity and visceral fat. It has even been shown that the abundance of this family increases in response to weight loss. With respect to dietary effects, an increase in Christensenellaceae has been observed in response to dietary interventions with prebiotic fibers (resistant starch, galactooligosaccharides, and polydextrose). It has also been associated with a diet that is low in refined sugars and high in fruit, vegetables, and animal protein. Interestingly, Christensenellaceae is a highly heritable taxon, often associated with host health [57].

Another bacterial group that was characteristic of our control group was the genus *Akkermansia*, which includes the species *Akkermansia muciniphila*. This bacterium has been negatively associated with obesity and metabolic dysfunction, as it promotes intestinal barrier integrity and prevents inflammation that is secondary to mucosal permeability [58]. Depommier et al. even demonstrated that supplementation with *A. muciniphila* orally for three months reduced insulin resistance, total cholesterol, body weight, and fat mass, as well as some markers of inflammation and liver dysfunction [59].

In general, we reaffirm that the microbial profile of our control group was polarized towards Gram-positive, butyrate-producing bacteria. These include *Coprococcus*, *Clostridium*, Actinomycetes, *Butyrivibrio*, and *Eubacterium hallii*. Butyrate has shown positive effects on the host, as it helps maintain intestinal barrier integrity, modulates immune responses, and participates in the synthesis of some vitamins, including vitamin B9 (folic acid). Moreover, in the context of obesity, butyrate supplementation has been shown to improve insulin sensitivity and decrease adiposity. Likewise, consuming a diet that is high in prebiotic fibers can promote the growth of bacterial groups that are capable of producing butyrate through the fermentation of non-digestible polysaccharides [40]. The Mediterranean diet, for example, which is high in fiber, promotes a mutualistic relationship between *Bifidobacterium adolescentis* and *Eubacterium hallii*; the latter is well known for its butyrate production [9].

We consider it important to highlight the presence of *Eubacterium hallii* in our study, since we found interesting correlations with some biochemical parameters: negative correlations with triglycerides and glucose and positive ones with HDL-c. In a previous study carried out by our research group in patients with HIV and MetS in western Mexico, we reported similar findings regarding *Eubacterium hallii*. This bacterial species displayed negative correlations with metabolic and inflammation markers, such as triglycerides, LDL-c, and C-reactive protein [36]. We theorize that *E. hallii* could be an interesting therapeutic target in cardiometabolic pathologies such as obesity and MetS.

Another bacterial genus that deserves special attention is *Coprococcus*. This genus is known for both its production of butyrate and of B vitamins, including B9 [56]. This is consistent with what we found in the PICRUSt2 analysis, which shows that the metabolic pathway for folate synthesis was increased in the control group and nearly depleted in the obese group. This is a novel finding, since the disruption of microbial folate metabolism pathways in obesity are described for the first time in universal research and is unprecedented in patients from western Mexico. Recently, Navarrete-Muñoz et al. demonstrated that a folate-rich diet is associated with lower basal glucose levels and higher HDL cholesterol concentrations in high-risk cardiometabolic subjects [60]. Additionally, in their systematic review, Köse et al. described that subjects with an elevated BMI have lower blood folate concentrations compared to individuals with a lower weight. Individuals who are overweight often eat unhealthy diets that are high in energy and low in fruits, vegetables, and dietary supplements, all of which could affect folate levels. Moreover, high body fat could reduce vitamin B9 absorption by the intestinal epithelium. Although the mechanism is not clearly elucidated, dietary folate deficiencies are associated with a higher lipid storage and leptin secretion of adipocytes [61].

Regarding vitamin B7, Belda et al., in their cross-sectional European MetaCardis study, reported alterations in microbial biotin (vitamin B7) metabolism, evaluated by metagenomic quantification in patients with severe obesity. These results correlated with suboptimal systemic biotin levels in these patients [62]. Biotin has been shown to be involved in glucose and lipid homeostasis [63]. Therefore, these interesting findings strengthen the clinical importance of knowing the bioavailability of vitamin B9 and B7 in patients with obesity, as well as how these vitamins impact the host’s metabolism. B-vitamin producing bacteria, along with their metabolites, could also be considered as future therapeutic targets.

Finally, we described the metabolic pathways that are specific to the Ob group’s microbiome and suggested an association with the clinical features of obesity and metabolic dysfunction. Mainly, twelve metabolic pathways were found to be significantly enriched in the obese group compared with the control. Of these pathways, six of them are involved in energy metabolism, mainly carbohydrate metabolism. Glycolysis, pyruvate fermentation to lactate, the pentose phosphate pathway, and the Krebs cycle (by acetate producers) pathways were found to be increased. This finding is in agreement with Duan et al., who found that subjects with obesity have abnormalities in their bacterial carbohydrate metabolism [64]. These metabolic pathways reflect the capacity of the intestinal microbiota to extract energy from the diet. Similarly, Chávez-Carbajal et al. concluded that obesity is associated with a bacterial profile with higher saccharolytic activity and a greater capacity to produce SCFAs. These pathways increase acetyl-CoA levels in the host, harvesting more energy from the diet and promoting dyslipidemia and adiposity [42]. Additionally, the Krebs cycle pathway, specifically that of acetate-producing bacteria, was also increased in the Ob group. This is consistent with Wan et al., who concluded that organic acids that are involved in the Krebs cycle, such as succinate and propionate, may be involved in the pathophysiology of obesity. For example, it is believed that propionate can stimulate glycogenolysis, hyperglycemia, compensatory hyperinsulinemia, and subsequent insulin resistance. Also, fecal propionate concentrations have shown a positive correlation with BMI. Moreover, succinate, which is a precursor of propionate, is also positively related to body weight. It is worth mentioning that a future line of research for our group is to study the fecal and systemic levels of SCFAs in patients with obesity, given their important implications in host metabolism.

Regarding the limitations of our study, due to the cross-sectional nature of the present research, causation cannot be inferred. Moreover, this was a single-center study with a relatively small sample size. Another limitation is that the study was conducted exclusively in the western Mexican population; therefore, results cannot be generalized to other regions of the world. As future lines of study, we consider evaluating additional parameters, mainly host-related factors such as lifestyle and diet, which will give greater strength to the present study.

## 5. Conclusions

Our study showed a strikingly different gut microbiota profile between subjects with and without obesity, regarding structure, taxonomy, and function. The obese group demonstrated a clear decrease in richness and diversity, as well as an aerotolerant microenvironment that was dominated by Gram-negative bacteria. We identified bacteria with potential proinflammatory and pathogenic effects, such as the Negativicutes class, and genera like *Escherichia*/*Shigella* and *Prevotella*. Likewise, an increase in short-chain fatty acid-producing bacteria was found, especially the genus *Lachnoclostridium.* In addition, there was a decrease in bacteria that are associated with metabolic health and the lean phenotype (Christensenellaceae, *Akkermansia*, and *Eubacterium hallii*). Interestingly, the PICRUSt2 analysis showed depletion of vitamin B9 metabolism and an increase in carbohydrate metabolism pathways. The IM of patients with obesity possesses a dysbiotic and proinflammatory environment, with an increased saccharolytic activity, all of which could contribute to lipogenesis and adiposity. These findings are described for the first time in the adult population of western Mexico. Further studies with a greater number of patients are needed in our population, in order to categorize subjects according to their obesity class and obtain a more accurate IM profile that may explain the development and evolution of this highly prevalent metabolic disease.

## Figures and Tables

**Table 1:** Demographic and biochemical characteristics of the recruited participants.

Characteristics	Ob Group (n = 38)	Control Group (n = 27)	*p*-Value
Age	37.7 ± 12.0	44.2 ± 12.4	0.088 ^a^
Sex			
Men, n (%)	17 (44.7%)	14 (51.9%)	0.571 ^c^
Women, n (%)	21 (55.3%)	13 (48.1%)	
BMI (kg/m^2^)	35.6 (32.8–42.7)	24.9 (23.1–26.9)	<0.001 *^b^
Body fat %	45.0 ± 7.9	29.3 ± 7.9	<0.001 *^a^
Glucose (mg/dL)	97.0 (92.0–105.3)	90.0 (85.5–94.5)	0.001 *^b^
Triglycerides (mg/dL)	213.2 ± 77.1	126.6 ± 78.3	0.001 *^a^
TC (mg/dL)	192.9 ± 28.6	158.6 ± 26.4	0.005 *^a^
LDL-c (mg/dL)	92.0 (88.5–115.5)	100.0 (59.0–113.0)	0.540 ^b^
HDL-c (mg/dL)	35.0 (28.0–46.0)	44.0 (40.0–59.0)	0.002 *^b^
SBP (mmHg)	135.9 ± 19.9	110.4 ± 20.0	0.002 *^a^
DBP (mmHg)	92.5 ± 11.0	77.3 ± 11.5	0.001 *^a^

Abbreviations: BMI (body mass index), SBP (systolic blood pressure), DBP (diastolic blood pressure), TC (total cholesterol), HDL-c (high-density lipoprotein cholesterol), LDL-c (low-density lipoprotein cholesterol). Data are expressed as average ± standard deviation, or as median and interquartile ratio (IQR). Statistical analysis: ^a^ Student’s *t*-test, ^b^ U of Mann–Whitney, ^c^ χ^2^. *p* < 0.005 is considered statistically significant. * Indicates statistically significant differences with respect to the control group.

## Data Availability

All data generated or analyzed during this study are included in this published article and are available by request, due to ethical restrictions.

## Supplementary Materials

The following supporting information can be downloaded at https://www.mdpi.com/article/10.3390/metabo14020121/s1: Figure S1: Scatter plot of Proteobacteria/Firmicutes, Anaerobic/Aerobic, Gram^+^/Gram^−^ in obesity (Ob) and without obesity (control) groups; Figure S2: Correlations between blood biochemical parameters and *Eubacterium hallii* in obesity (Ob) and without obesity (control) groups; Figure S3: Gel electrophoresis of PCR of V3-V4 regions from 16S rRNA.
