# Supplementary material for: Intestinal Dysbiosis in Subjects with Obesity from Western Mexico and Its Association with a Proinflammatory Profile and Disturbances of Folate (B9) and Carbohydrate Metabolism
Source: Metabolites. 2024 Feb 11;14(2):121. doi: 10.3390/metabo14020121 (PMC10890169; doi:10.3390/metabo14020121)
Supplement: Supplementary file 1 [file metabolites-14-00121-s001.zip › V2-Supplementary_material_Obesity_metabolites.pdf]

Supplementary material: Intestinal dysbiosis in obese subjects from western Mexico and its association with a proinflammatory profile and disturbances of folate (B9) and carbohydrate metabolism

Verónica Riggen-Bueno [1,2], Susana Del Toro-Arreola [3], Tonatiuh Abimael Baltazar-Díaz* [3], Alejandra N. Vega-Magaña [4], Marcela Peña-Rodríguez [5], Paula Alejandra Castaño-Jiménez [3], Laura Verónica Sánchez-Orozco [3], José María Vera-Cruz [6], Miriam Ruth Bueno-Topete* [3]

[1]Servicio de Nutrición Clínica, Hospital Civil de Guadalajara, Unidad Hospitalaria Fray Antonio Alcalde

[2] Maestría en Nutrición Clínica, Universidad del Valle de Atemajac

[3] Instituto de Investigación en Enfermedades Crónico Degenerativas, Universidad de Guadalajara

[4] Instituto de Investigación en Ciencias Biomédicas, Universidad de Guadalajara

[5] Laboratorio de Diagnóstico de Enfermedades Emergentes y Reemergentes, Universidad de Guadalajara

[6] Instituto de Nutrigenética y Nutrigenómica Traslacional, Universidad de Guadalajara

1. Supplementary figures

1.1 Supplementary figure S1

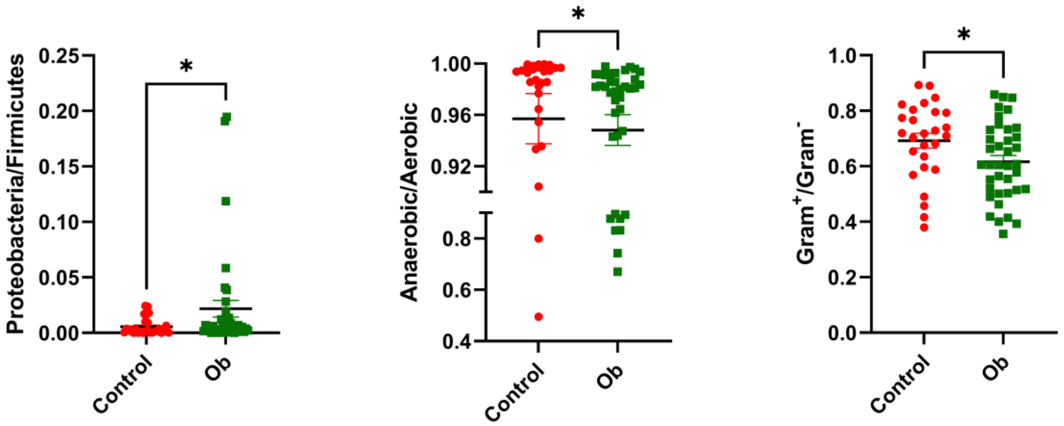

Figure S1: Supplemental figure 1. Scatter plot of Proteobacteria/Firmicutes, Anaerobic/Aerobic, Gram+/Gram- in obesity (Ob) and without obesity (control) groups. Data were first transformed by means of centered log-ratio (clr). Then, relative abundances were obtained and calculated according to material and methods section. Results are showed as mean ± SEM. Analysed by Mann–Whitney U test with Benjamini-Hochberg (BH) multiple testing correction. * $p < 0.05$.

1.2 Supplementary figure S2

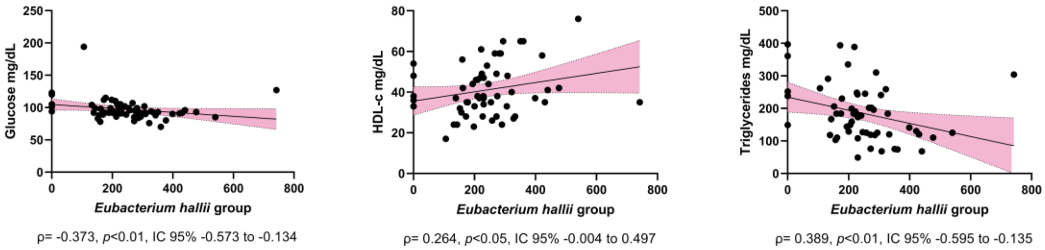

Figure S2: Supplemental figure 2. Correlations between blood biochemical parameters and *Eubacterium hallii* in obesity (Ob) and without obesity (control) groups. Spearman's ρ (rho) and p-values (two-tailed) are showed below each diagram. Glucose, triglycerides and high density lipoproteins (HDL-c). Values on the X axis are expressed as clr-transformed total abundances.

1.3 Supplementary figure S3

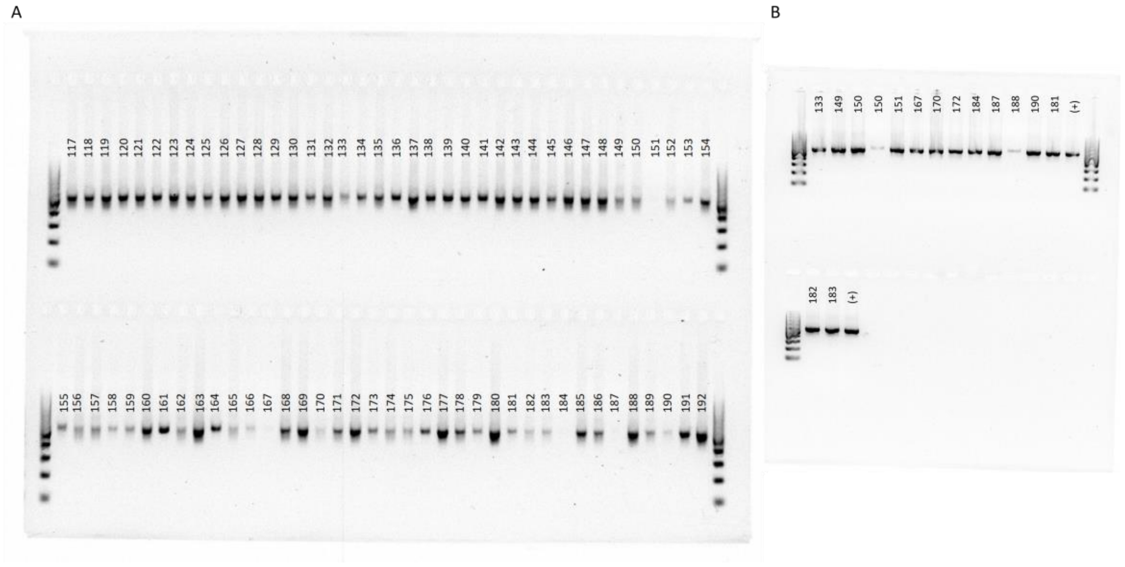

Figure S3: Supplemental figure 3. A. Gel electrophoresis of PCR of V3-V4 regions from 16S rRNA using primers with adaptors, as described in methodology section. Lanes 117-192 represent each sample. Those samples from which no amplicon was obtained, or it was obtained in low concentrations, were repeated, showing them on gel. B. Gel electrophoresis from first and last lanes from upper and lower sections of the gel are 100 pb DNA ladder.

Supplementary data S1

Feature statistics and representative sequences have been deposited in the figshare repository, with the following DOI: 10.6084/m9.figshare.25135706
